# Protocatechuic acid, ferulic acid and relevant defense enzymes correlate closely with walnut resistance to *Xanthomonas arboricola* pv.* juglandis*

**DOI:** 10.1186/s12870-022-03997-9

**Published:** 2022-12-20

**Authors:** Qian Zhang, Meixuan Li, Guiyan Yang, Xiaoqiang Liu, Zhongdong Yu, Shaobing Peng

**Affiliations:** 1 Laboratory of Walnut Research Center, College of Forestry, Northwest A & F University, Shaanxi 712100 Yangling, China; 2 Department of Foreign Languages, Northwest A & F University, Shaanxi 712100 Yangling, China

**Keywords:** *Juglans regia* L., *Xanthomonas*, Disease resistance, Defense enzyme, Phenolic compounds

## Abstract

**Background:**

*Juglans regia* L. is an important nut tree that has a wide range of distribution in temperate regions of the world. In some walnut orchards, walnut blight can become a problematic disease that affects the growth of walnut trees. To explore the correlation between biochemical response and walnut resistance, we inoculated four walnut cultivars with *Xanthomonas arboricola* pv. *juglandis* (Xaj). The walnut cultivars were, namely, ‘Xiangling’, ‘Xiluo 2’, ‘Yuanfeng’ and ‘Xifu 2’. Total phenol content (TPC) and total flavonoid content (TFC) were measured, whereby nine major phenolic compounds and several relevant enzymes were identified.

**Results:**

The results showed that the most resistant and susceptible walnut varieties were ‘Xiluo 2’ and ‘Xifu 2’ respectively. The reaction of walnut to Xaj was characterized by the early accumulation of phenolic compounds in the infected site. After inoculation with Xaj, we found that the resistant variety ‘Xiluo 2’ show the significant differences with other varieties at different time points through the determination of related antioxidant enzymes such as catalase (CAT) and peroxidase (POD). Meanwhile, the phenylalanine ammonia lyase (PAL) of ‘Xiluo 2’ increased significantly at 8 day post infection (dpi) and made differences from the control samples, while other varieties changed little. And the polyphenol oxidase (PPO) was significantly higher than in the control at 16 dpi, maintaining the highest and the lowest activity in ‘Xiluo 2’ and ‘Xifu 2’ respectively. It was also found that the content of protocatechuic acid in all cultivars increased significantly at 4 dpi, and ‘Xiluo 2’ was significantly higher than that of the control. In the early stage of the disease, ferulic acid content increased significantly in ‘Xiluo 2’.

**Conclusion:**

Our findings confirmed that the metabolism of phenolic compounds and related defense enzymes are of great significance in the response of walnut to Xaj.

**Supplementary Information:**

The online version contains supplementary material available at 10.1186/s12870-022-03997-9.

## Introuction

*Juglans regia* L. is one of the most important economic tree species of the Juglandaceae family. Walnut is cultivated worldwide for its delicious taste and rich nutritional qualities, with its protein and protein hydrolysates that play an important role in promoting health [1]. Among the pathogens that affect walnut, there is a species of bacteria, namely, *Xanthomonas arboricola* pv. *juglandis* (Xaj), which causes walnut blight. It is particularly damaging in wet years and on early leafing cultivars, as it poses a serious threat to walnut growth and production. It can enter through stomata, lenticels, and stigmas, and then spread by the wind and rain, or by insects and agricultural operations [2–5]. The occurrence of *Xanthomonas* has reportedly affected rice [6], citrus [7], walnut and other plants. To date, the control of walnut blight disease has relied on copper-based biocides [8]. Nonetheless, biocides may be unsustainable development because of their toxicity, their impact on the environment and on natural ecosystems [9]. The most economical, effective measure for the control of this disease is to cultivate blight-resistant walnut cultivars.

Plant resistance can be classified into two categories: constitutive resistance and inducible resistance. Constitutive resistance is an inherent trait that relies on epidermal wax, cell walls, and cuticles [10, 11]. Inducible resistance, on the other hand, refers to a set of induced defense responses evolved by plants as a result of pathogen stimulation, whereby pathogens are resisted by plants through better mechanisms of defense. This active defense-response can strengthen the endogenous defense ability of plants which comprises the accumulation of ROS, rapid changes in the biochemical features of cell walls and the enhancement of various defense-related enzyme activities [12]. The interactions between walnuts and disease involve the coordination of ROS and other signals, as these are dependent on stress-specific chemicals that initiate self-protective responses in neighboring cells [13]. Previous works have summarized that plants respond to pathogenic attacks by staging a transient burst of ROS which triggers a chain of reactions that play a protective role at times of infection by inhibiting the growth of pathogens. RBOHs, a protein complex, are playing a key role in the network of ROS production. Specifically, it transfers electrons from the NADPH in the cytosolic fluid to oxygen in the apoplast (i.e. the main source of ROS) in order to generate superoxide anion (O_2_.^−^). The latter is then converted to H_2_O_2_, either spontaneously or by SOD. As SOD produces H_2_O_2_, the disproportionate amount of H_2_O_2_ is then degraded by POD and CAT into non-toxic H_2_O_2_ and oxygen, thereby protecting plant cells from serious oxidative damage [14, 15].

Phenols are a pervasive group of secondary metabolites in plants [16]. Gallic acid, ferulic acid and quercetin are different types of phenolic chemicals in walnuts [17]. Phenolic chemicals comprise a part of inducible resistance. In plant defense, phenolic compounds can act as physical or chemical barriers against pathogenic invasion. Previous research indicated a strong linear relationship between phenolic compounds in walnut microshoots and antioxidant activity [18]. Moreover, a detailed research on plant-pathogen interactions revealed that these phenolic compounds are generated through the phenylpropanoid metabolic pathway, with the first reaction being catalyzed by PAL. It can guide carbon to flow to phenolic compounds, followed by the synthesis of lignin, phytoalexin and flavonoids to participate in the defense process of plants against pathogens [19]. It was reported that the differential expression of phenolic metabolic pathway enzyme activity is one of the main reasons for the differences in resistance among varieties [20]. PPO has been observed to have roles in the phenylpropane pathway. It is a copper-containing enzyme that inhibits pathogens by converting monophenols into o-catechol compounds with oxygen as a substrate. Khodadad et al. implied that the walnut-bacterial blight interaction induced *JrPPO-1* and the activity of PPO after inoculating Xaj [21, 22].

Identification and utilization of disease resistance may provide a basis for a feasible control strategy for walnut blight. Although walnuts are rich in phenolic compounds, the potential for disease resistance by phenolic chemicals is not fully understood. Therefore, the differences in phenolic metabolism of different walnut cultivars after infection were detected by HPLC in our research. In addition, the changes of activities in defense enzymes were studied in the process of infection in this study. This study provided valuable physiological information for understanding the response of walnut to *Xanthomonas arboricola* pv. *juglandis*, and revealed how relevant defense-related enzymes and chemicals can be important in the resistance against blight disease, thereby hinting at possible ways into the breeding of resistant cultivars.

## Results

### Field investigation and artificial inoculation on the four walnut cultivars

**Fig. 1:**
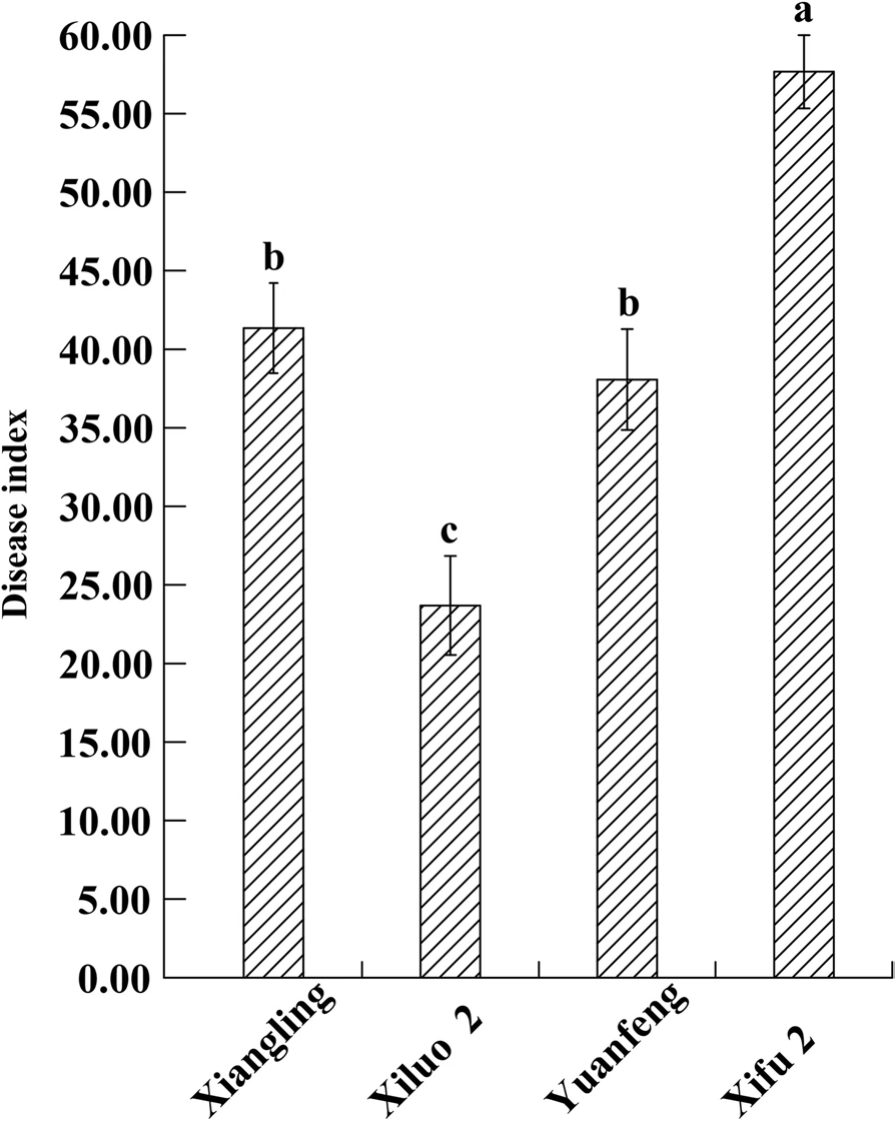
The disease index and resistance evaluation of different cultivars in the field to walnut blight

**Fig. 2:**
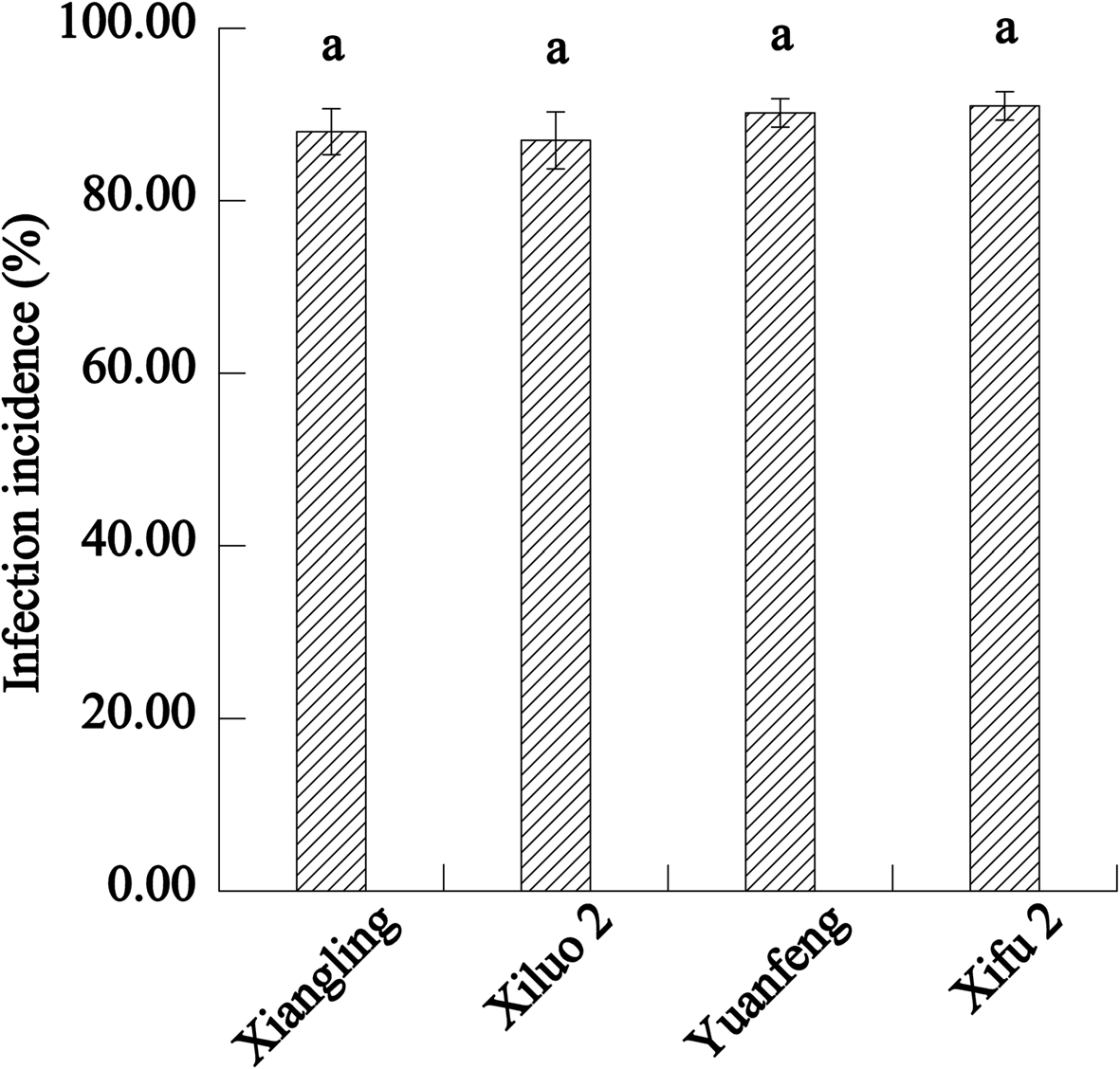
Infection incidence of four cultivars at 16 day post infection (dpi). The values are expressed as the mean ± standard deviation of three biological replicates. Letters indicated significant differences at a level of *p* < 0.05

**Fig. 3:**
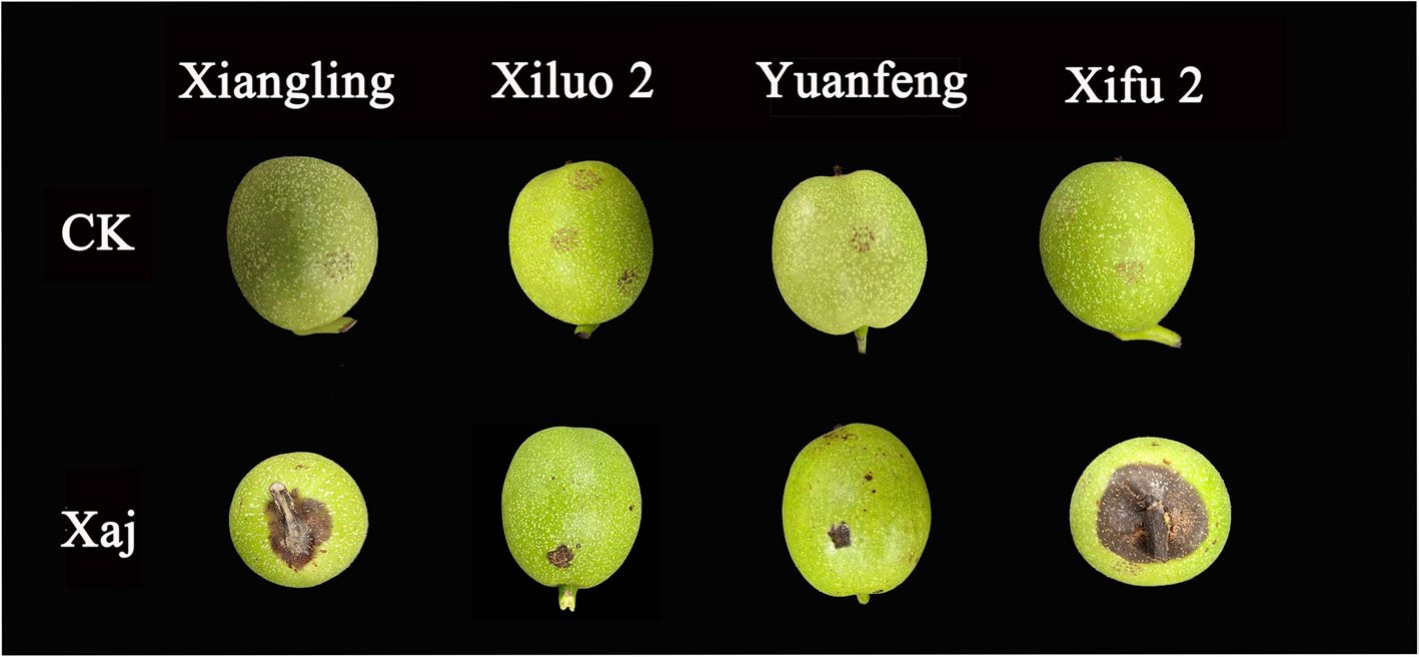
Phenotypic observation of different varieties 16 days after inoculation

**Fig. 4:**
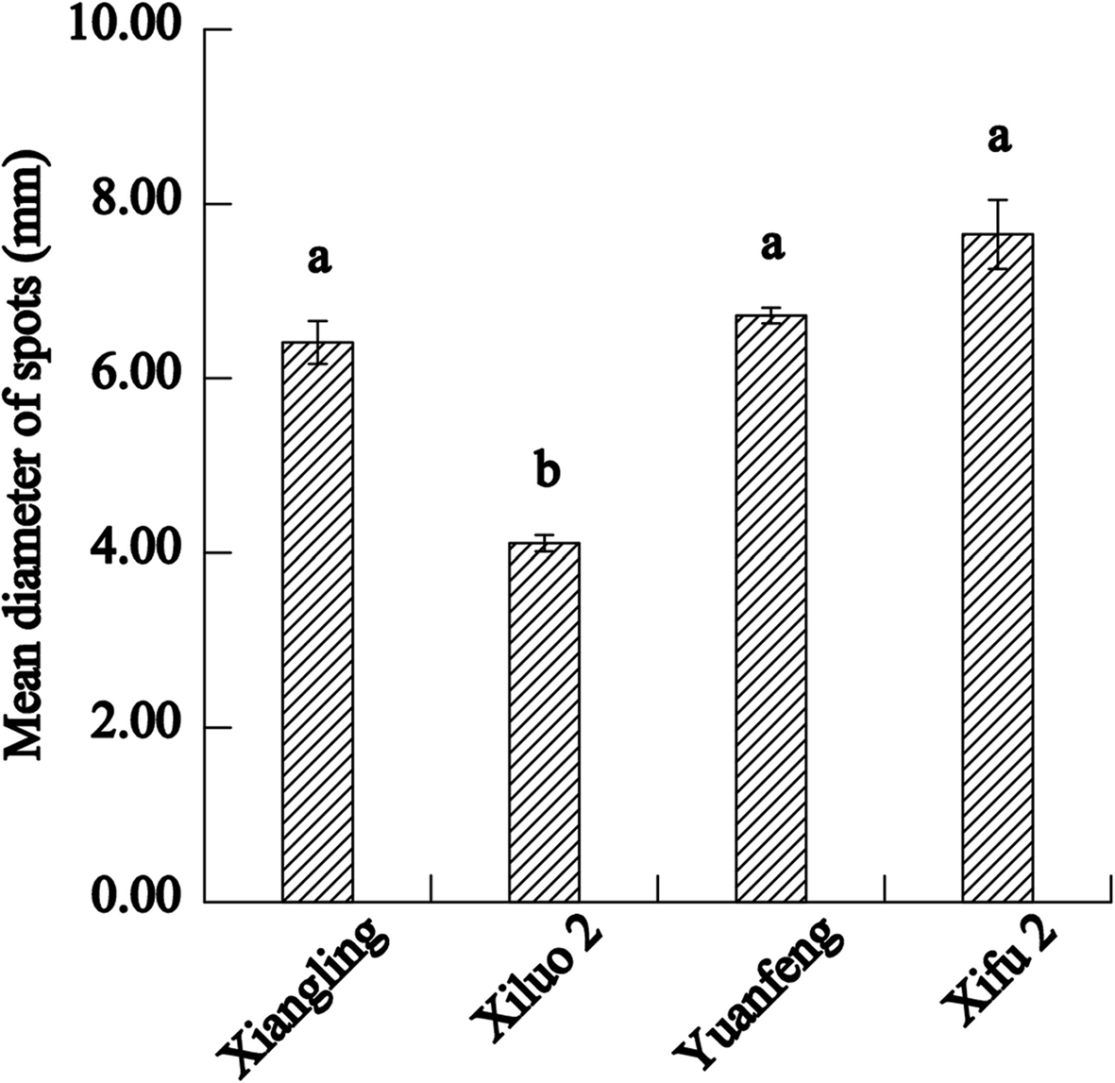
Average lesion caused by Xaj in fruits at 16 dpi. The values are expressed as the mean ± standard deviation of three biological replicates. Letters indicated significant differences at a level of *p* < 0.05

The field investigation results of four walnut varieties showed that the disease resistant and the susceptible variety were ‘Xiluo 2’ and ‘Xifu 2’, respectively (Fig. 1). On the other hand, we proved that all cultivars were successfully infected with Xaj by artificial inoculation and showed different levels of susceptibility. Briefly, the incidence rate of blight disease was most prevalent (by more than 85%) in each cultivar at 16 dpi (Fig. 2). Meanwhile, the sizes of fruit rot and blackening were varied from all four cultivars at 16 dpi (Fig. 3). The average diameter of lesions on the fruits of different walnut cultivars after inoculation with Xaj was used as a measure of walnut resistance against pathogenic activity. At 16 dpi, the difference of lesion size between ‘Xiluo 2’ and ‘Xifu 2’ was the largest, meaning that ‘Xifu 2’ was the most susceptible, whereas ‘Xiluo 2’ was the most resistant (Fig. 4).

### Antioxidant activity

**Fig. 5:**
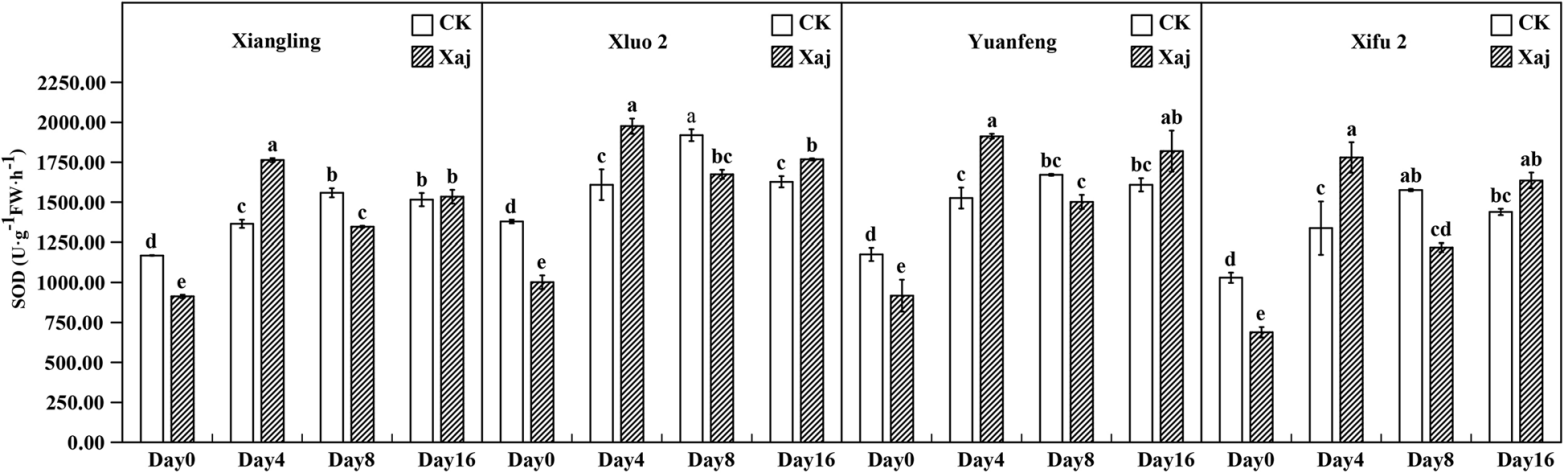
Changes in SOD activity in the control (CK) and inoculated walnut fruits (Xaj) from 0 to 16 days. And the values are expressed as the mean ± standard deviation of three biological replicates. Letters indicated significant differences at a level of *p* < 0.05

After inoculation with the pathogen, SOD activity increased at the outset but then decreased. This pattern of change was observable in all four cultivars. Initially, the SOD activity of all cultivars infected with the pathogen was significantly lower than that of the control. At 4 dpi, the SOD activity in all cultivars staged an obvious increase and was significantly higher than in the control fruits. At 16 dpi, there was no significant difference between the control and the inoculated samples of three cultivars, except that the SOD in ‘Xiluo 2’ was significantly higher than that of the control (Fig. 5).

**Fig. 6:**
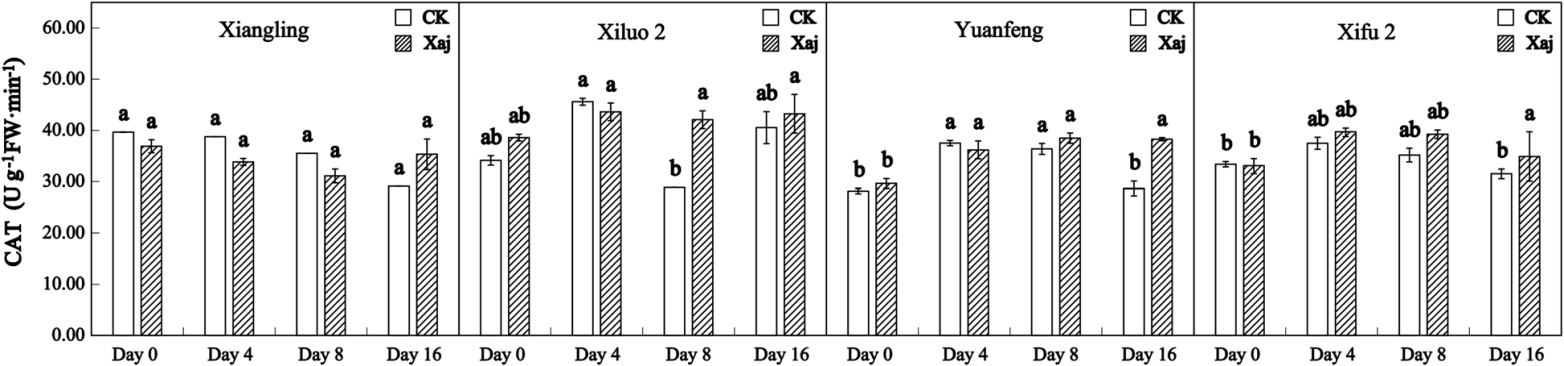
Changes in CAT activity in the control (CK) and inoculated walnut fruits (Xaj) from 0 to 16 days. And the values are expressed as the mean ± standard deviation of three biological replicates. Letters indicated significant differences at a level of *p* < 0.05

CAT activity of all varieties after inoculation was basically similar to the control in the whole process. However, there were some differences among the varieties. Specifically, at 8 dpi, the CAT activity in ‘Xiluo 2’ was significantly higher than in the control, whereas differences in the CAT activities of ‘Xifu 2’ and ‘Yuanfeng’, occurring between the inoculated fruits and the control fruits, were observed at 16 dpi. This result showed that CAT responds earlier in the resistant variety (Fig. 6).

**Fig. 7:**
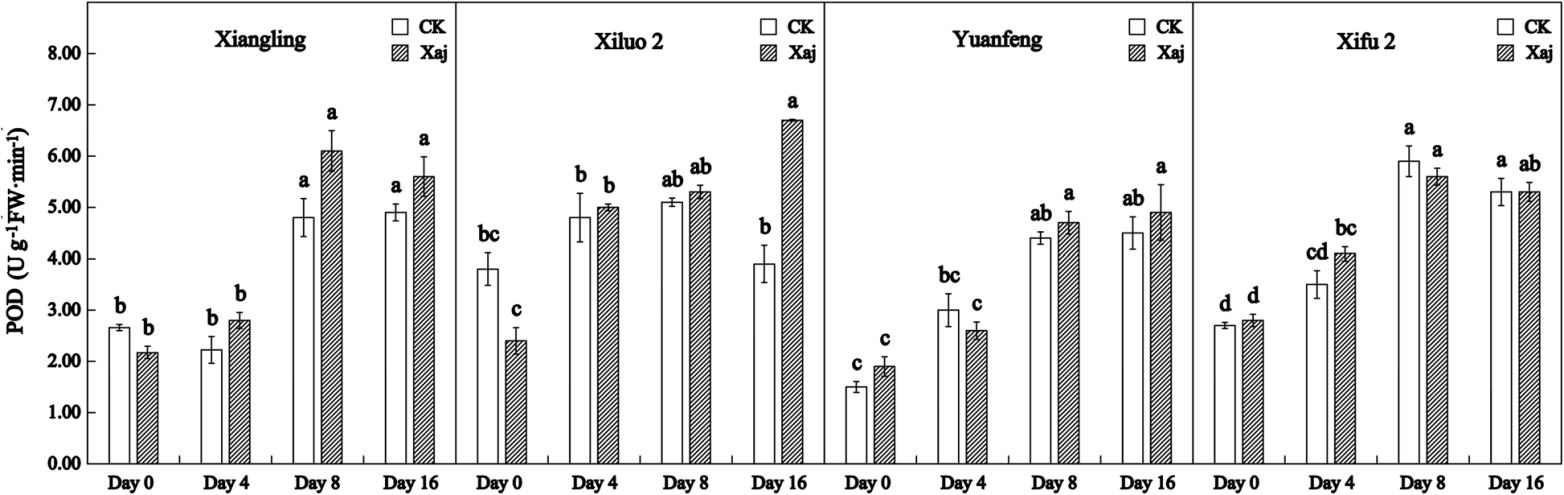
Changes in POD activity in the control (CK) and inoculated walnut fruits (Xaj) from 0 to 16 days. And the values are expressed as the mean ± standard deviation of three biological replicates. Letters indicated significant differences at a level of *p* < 0.05

POD activity showed an upward trend in all cultivars between the control and inoculation samples. The enzyme activity of ‘Xiluo 2’ increased rapidly at 4 dpi. And the other three varieties began to increase rapidly at 8 dpi. However, there was no significant difference in POD activity between the control and inoculation fruits in the first 8 days of the four varieties. The POD activity of ‘Xiluo 2’ still showed higher activity and was significantly higher than that of the control fruits at 16 dpi, while the other varieties changed little (Fig. 7).

### PAL activity analysis

**Fig. 8:**
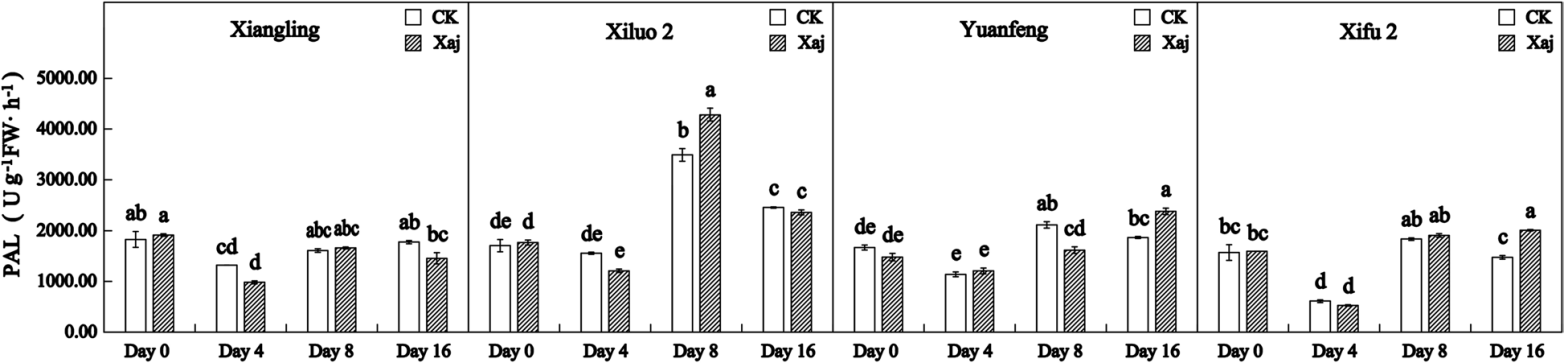
Changes in PAL activity in the control (CK) and inoculated walnut fruits (Xaj) from 0 to 16 days. And the values are expressed as the mean ± standard deviation of three biological replicates. Letters indicated significant differences at a level of *p* < 0.05

At 4 dpi, PAL activity decreased in all cultivars and, in particular, it decreased in ‘Xifu 2’ by nearly 2-fold. And the PAL activities of control samples were similar to the inoculation. At 8 dpi, an obvious increase in PAL activity was observed. The PAL activity of walnut fruits infected with Xaj was significantly different from the fruits treated with distilled water. Meanwhile, there were negligible differences in other cultivars. At 16 dpi, inoculated samples of ‘Yuanfeng’ and ‘Xifu 2’ showed significant levels of PAL activity, compared to the control (Fig. 8).

### PPO activity analysis

**Fig. 9:**
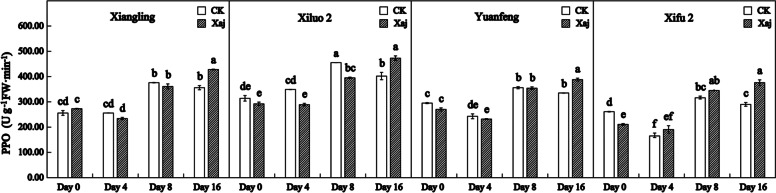
Changes in PPO activity in the control (CK) and inoculated walnut fruits (Xaj) from 0 to 16 days. And the values are expressed as the mean ± standard deviation of three biological replicates. Letters indicated significant differences at a level of *p* < 0.05

PPO took a trend of increase in activity, but with variable intensities among the cultivars. At 8 dpi, PPO activities of ‘Xiangling’, ‘Yuanfeng’ and ‘Xifu 2’ were significantly increased both in the control and inoculation walnut fruits. But there was no significant difference between them. At 4 and 8 dpi, however, PPO activity of ‘Xiluo 2’ in the control fruits was significantly higher than that of the inoculation. And PPO activity in inoculated fruits was significantly higher than that of the control at 16 dpi in all varieties (Fig. 9).

### Phenolic chemical content analysis

#### Changes in the content of TPC and flavonoids TFC

**Fig. 10:**
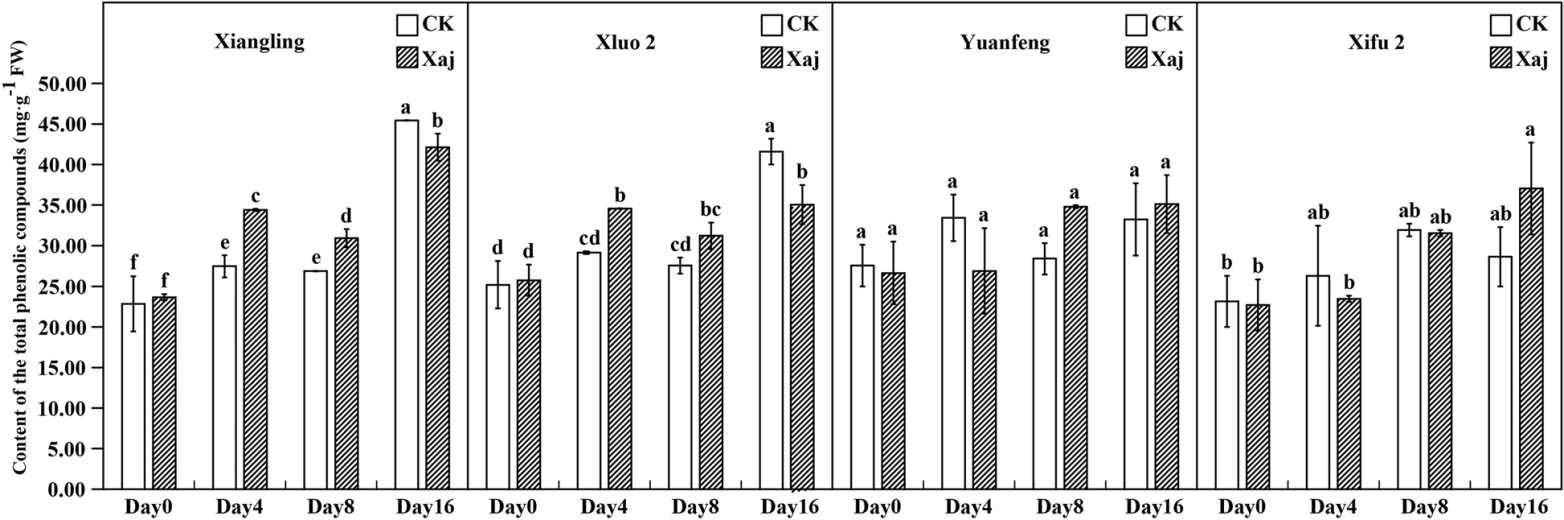
The total phenolic compounds (TPC) content in the control (CK) and inoculated walnut fruits (Xaj) during 0–16 days. And the values are expressed as the mean ± standard deviation of three biological replicates. Letters indicated significant differences at a level of *p* < 0.05

The changes in TPC were mainly reflected in ‘Xiangling’ and ‘Xiluo 2’, while no changes were observed between ‘Yuanfeng’ and ‘Xifu 2’. Phenolic contents increased in ‘Xiangling’ and ‘Xiluo 2’ at 4 dpi, while the TPC of inoculated fruits was significantly higher than in the control. At 16 dpi, although the TPC increased rapidly and reached a maximum value, phenolic contents in control samples were significantly higher than those in inoculated samples (Fig. 10).

**Fig. 11:**
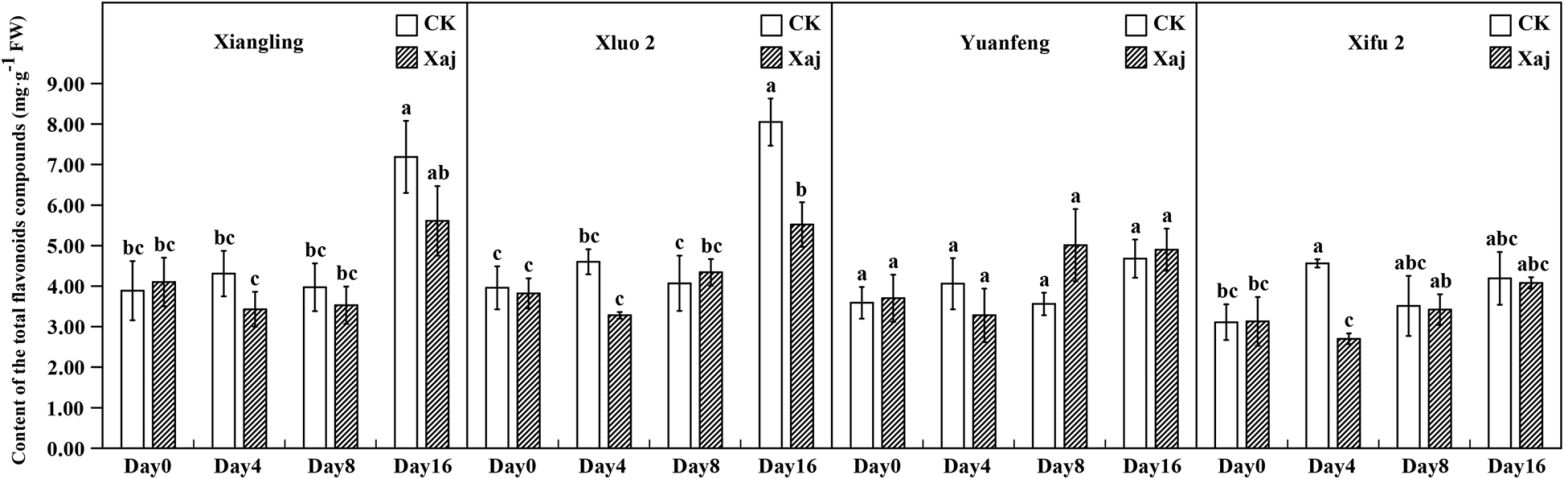
The total flavonoids compounds (TFC) content in the control (CK) and inoculated walnut fruits (Xaj) during 0–16 days. And the values are expressed as the mean ± standard deviation of three biological replicates. Letters indicated significant differences at a level of *p* < 0.05

The content of TFC was far less than in the TPC. At 4 dpi, the TPC in variety ‘Xifu 2’ showed that the control was significantly higher than in the inoculation fruits. At 16 dpi, the TFC in ‘Xiluo 2’ and ‘Xiangling’ were also significantly lower than that of the control (Fig. 11).

### Analysis of monomer phenol contents

After treatments, the contents of catechin, *p*-coumaric acid, gallic acid, protocatechuic acid, chlorogenic acid and ferulic acid were detected by liquid chromatograph in the four cultivars. Some of these played a role in interactions between walnut fruits and pathogenic activity.

The content of gallic acid in ‘Xiangling’ decreased as a whole both in control and inoculation fruits. But there was no significant difference between them. And we also found that, except at 8 dpi, the content of gallic acid in ‘Xiluo 2’ was significantly higher in control compared to inoculation fruits ( Figure S1).

At 4 dpi, the catechin content in ‘Xiangling’ and ‘Xiluo 2’ increased by 140% and 7.4-fold, respectively, and then decreased to the initial content. The variety ‘Xiluo 2’ with inoculated Xaj of the catechin contents was significantly lower than that of the control fruits (Figure S2). Furthermore, the content of chlorogenic acid decreased rapidly in the ‘Xifu 2’ with Xaj at 4 dpi. And it was very similar to the control. The other three cultivars showed no changes in the whole experiment. This observation suggested a weak interaction between chlorogenic acid and the pathogen (Figure S3). The amount of *p-*coumarin in ‘Xiangling’ and ‘Yuanfeng’ increased in the later stage of inoculation and was significantly higher than in the control (Figure S4).

**Fig. 12:**
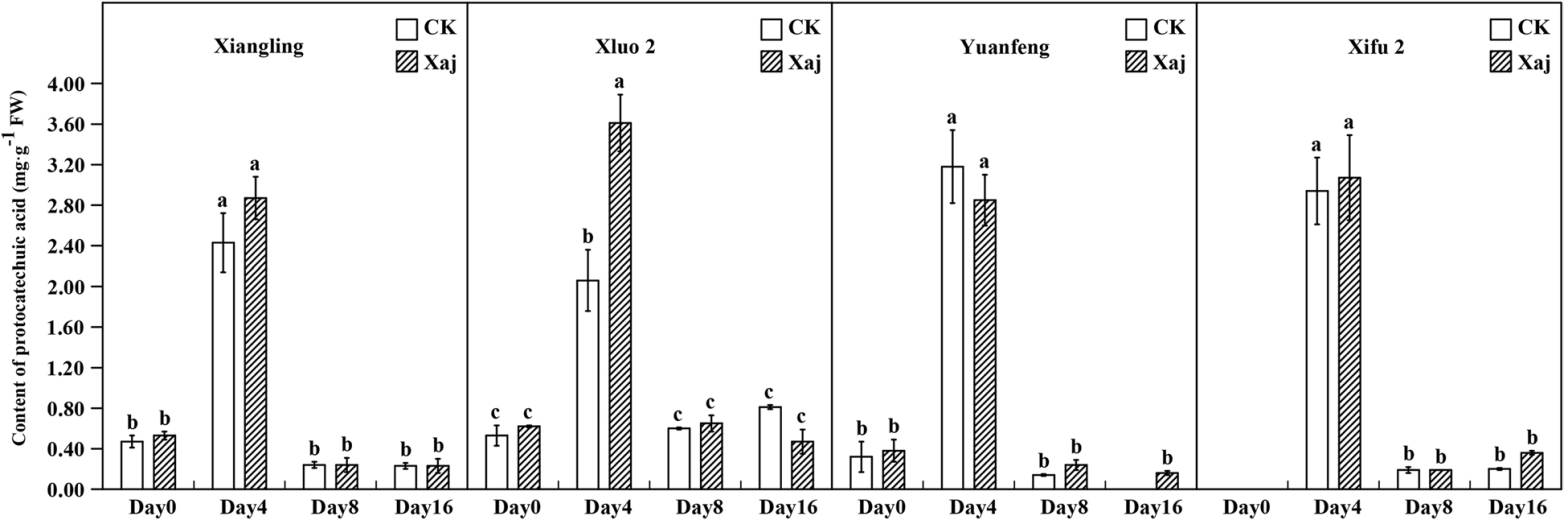
Protocatechuic acid content in the control (CK) and inoculated walnut fruits (Xaj) during 0–16 days. The blank histogram indicates that the content is too weak to detect. And the values are expressed as the mean ± standard deviation of three biological replicates. Letters indicated significant differences at a level of *p* < 0.05

**Fig. 13:**
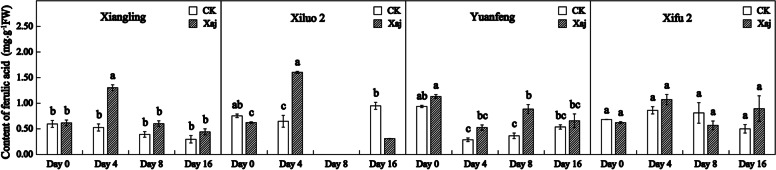
Ferulic acid content in the control (CK) and inoculated walnut fruits (Xaj) during 0–16 days. The blank histogram indicates that the content is too weak to detect. And the values are expressed as the mean ± standard deviation of three biological replicates. Letters indicated significant differences at a level of *p* < 0.05

Although the content of protocatechuic acid and ferulic acid was lower than other phenolic chemicals, their roles cannot be overlooked. At 4 dpi, protocatechuic acid increased significantly in all cultivars. And in the inoculation samples of ‘Xiluo 2’ variety was significantly higher than that of the control. However, there were no significant differences in the other three varieties. At 8 dpi, it decreased significantly to the initial content (Fig. 12). On the other hand, at 4 dpi, ferulic acid content increased substantially in the fruits of ‘Xiangling’ and ‘Xiluo 2’, and the difference was significant compared with the control fruits. At the same time, inoculated samples of ‘Yuanfeng’ was significantly higher than that of the control at 8 dpi (Fig. 13).

## Discussion

Despite their high monetary potential in production and nutritional value, walnut trees are susceptible to the occurrence of walnut blight disease which can restrict their economic output on a large scale. An illustration of the mechanism by which the walnut-pathogen interaction takes place can make significant contributions to basic research and breeding practice. In the current research, a series of changes were observed in the biochemical indexes of the four cultivars after exposure to pathogenic infection.

Phenolic compounds are found primarily in the cytoplasm and are separated from phenolase by the cell membrane system, meaning that phenolase does not come into direct contact with phenolic compounds. When plants are invaded by pathogens, the cell membrane system is destroyed and, thus, phenolic compounds can be released. This makes them have toxic effects through their conversion to phytoalexins and free radicals that originate in lignin precursors [23]. It has long been realized that the response of plants to pathogens is featured by the early accumulation of phenolic compounds at the infection site, and that pathogens develop limitedly [24]. In our results, the TPC in ‘Xiangling’ and ‘Xiluo 2’ increased significantly at 4 dpi. This was consistent with the significant increase of phenolic compounds in walnut inoculated with Xaj by Jiang et al. [25]. This phenomenon can explain that plant defense in walnut reacts to pathogens by an early accumulation of phenolic compounds. Phenolic accumulation is an initial response to inoculated pathogens and may reflect an increase in relatively nontoxic secondary metabolites, which eventually serve as precursors that bring on plant resistance [26]. In this regard, our results showed a remarkable accumulation of protocatechuic acid at 4 dpi in both resistant and susceptible cultivars.

On further analysis, the content of protocatechuic acid in ‘Xiluo 2’ was significantly higher than that of the control at 4 dpi. According to previous findings by Liu et al. [27], protocatechuic acid effectively inhibited the growth of pathogens in the said research. Furthermore, protocatechuic acid is reportedly a precursor to gallic acid [28]. Briefly, protocatechuic acid is the product of 3-dehydroashikimic acid under the action of 3-dehydroashikimic acid dehydrogenase. Then it is hydroxylated to gallic acid by protocatechuic acid hydroxylase. It was reported that gallic acid can effectively inhibit the growth of pathogen that invade plants. However, in our experiment, the content of gallic acid after inoculation was similar to the control fruits in all varieties. This result may be caused by the low concentration of protocatechuic acid, resulting in the direct metabolic synthesis of gallic acid from 3-dehydrogenated shikimic acid [29, 30].

The accumulation of phenols in the infection site, caused by pathogenic bacteria, not only strengthened the cell wall structure but was also accompanied by the accumulation of reactive oxygen species, which promoted cross-linking reactions within the cell wall. A large number of studies have shown that the POD activity is positively correlated with the resistance of host plants. For example, in the study of the relationship between common bean and *Xanthomonas axonopodis* var. *fuscans*, higher POD activity was recorded in the pathogen infiltrated cotyledonary leaves than in control but greater activities occurred for the resistant cultivar than for the susceptible cultivar [31]. As shown in Fig. 7, POD activity increased rapidly in the resistant variety ‘Xiluo 2’ at 4 dpi until the late stage of disease development. Besides, POD is not only the primary enzyme for H_2_O_2_ decomposition, but it can also catalyze the dimerization of ferulic acid. Ferulic acid has long been considered to be an important component of the cell walls of all major vascular plants. When the pathogen invades, the occurrence of free radical coupling, as a reaction that involves ferulic acid residues, can strengthen the early structure of cell walls in association with phenolic cross-linking of hemicellulose via dicarboxylic acid. This is followed by the synthesis of phenolic monomers or by lignification which contributes to plant resistance against disease [32, 33]. In our study, the activity of POD and ferulic acid content of ‘Xiluo 2’ increased significantly at 4 dpi, which indicates that this variety can respond to Xaj through the catalysis of ferulic acid by POD.

PAL is a general measure of phenolic synthesis after infection. Radix et al. [34] confirmed that the involvement of metabolites of polyphenols in phenylpropane biosynthesis relates closely to walnut resistance against *Xanthomonas campestris* pv. *Juglandis*. The enzymatic step of this pathway is the conversion of L-phenylalanine to cinnamic acid by PAL, followed by *p*-coumaryl-CoA. On the one hand, the *p*-coumaryl-CoA further synthesizes phenolic acids, and on the other hand, it generates flavonoids under the catalysis of chalcone synthase (CHS) [35, 36]. Our study found that the content of the TFC was far less than that of the TPC. It can be speculated that the competitiveness of flavonoids biosynthesis was weakened, which promoted the effect of phenolic acids on walnut resistance to Xaj. Since the *VqMYB154* gene reportedly increased *STS* expression in wild grapes, plant interaction with pathogens caused a decrease in *CHS* competitiveness and promoted stilbene accumulation, thereby assisting plant resistance [37]. This also explains that there is no significant change in the determination of the TFC in our experiment. Previous research showed that pathogenic activity can lead to the accumulation of salicylic acid in infected tissues and, moreover, a role exists between PAL activity and pathogen-induced salicylic acid formation in many plants [38]. The resistance caused by salicylic acid against walnut blight can be studied further.

It was reported that the up-regulation of PPO changes the metabolism of phenylpropane pathway, leading to the accumulation of defensive phenolic compounds in plant cells, and thus strengthening the resistance of plants to pathogens [39]. The number of PPO genes varies from plant to plant. There is only a single PPO gene in walnut. PPO silencing leads to significant changes in the metabolism of phenolic compounds and their derivatives, such as coumaric acid and catechin, and the expression of phenylpropane pathway genes [40]. PPO is a nuclear-encoded and plastid copper-containing enzyme that catalyzes the oxidation of oxygen-dependent phenolic compounds to *o*-diquinones. Its quinone products are highly-active molecules that can modify and crosslink nucleophilic molecules covalently against pathogens. The highest expression levels of PPO are usually recorded in young tissues such as new leaves and fruits, which are particularly vulnerable to disease. According to a relevant study, walnut blight disease induced the expression of *JrPPO1* and PPO activity [21]. As shown in Fig. 9, PPO activity in all infected plants became significantly higher than that of the control at 16 dpi, suggesting that PPO may be an indirect regulator of cell death in walnut and may result in necrotic spots on the fruits [25]. At the same time, it can play a series of defense-related functions with ROS. At the time of pathogenic invasion, the direct toxicity of quinone mediated covalent modification in macromolecules and caused the direct antimicrobial toxicity of H_2_O_2_ which limited the progress of the disease. The cross-linking of oxidative phenols and the formation of lignin tend to produce a physical barrier against pathogens [41]. The accumulation of ROS in resistant tissues is considered to be the main mechanism of killing or inhibiting pathogens [42]. Among the different types of ROS, superoxide (O2.^−^) and H_2_O_2_ are involved functionally in plant resistance against numerous diseases [43]. CAT is one of the primary systems in plants that can enable the enzymatic hydrolysis of H_2_O_2_ molecules. Plants are affected by the outbreak of ROS against pathogens in different periods. They may have shown the symptoms when the ROS massively accumulate. For example, once the infected tissue begins to decay, expand or even sink, the active oxygen cannot prevent the pathogen from invading the host plant [20]. In our research, although the induction of CAT in all cultivars found little to no significant differences between the control and the infection samples during the whole test period, there are still some findings. Our studies showed that, at 8 dpi, CAT activity in ‘Xiluo 2’ was significantly higher than in the control, whereas differences in ‘Xifu 2’ and ‘Yuanfeng’ were observed at 16 dpi. This result showed that CAT responds earlier in resistant variety. SOD is known to function in association with ROS and, thus, can act against plant disease [43]. In our research, at 4 dpi, SOD activity in the inoculation fruits of all varieties was significantly higher than that of the control, indicating that the increase of SOD activity protected plant cells and played a role in the process of walnut resistance to Xaj at the initial stage of inoculation.

## Conclusion

Taken together, a resistant walnut cultivar was identified against *Xanthomonas*. By determining the biochemical reactions associated with plant responses to the pathogen, the results showed that there are different changes in the related defense enzymes and some phenols between the disease-resistant variety and other varieties. It suggested that they play a certain role in the interaction between walnut and Xaj. Our results provided important information on physiological aspects of walnut that induce resistance to walnut blight. Since reactions between the host and pathogenic microorganisms are highly complicated, it is recommended that future studies consider how a series of phenolic compounds can mediate plant resistance in the metabolic process through the phenylpropane pathway.

## Methods

### Experimental site and plant material

This study used four walnut cultivars: ‘Xiangling’, ‘Xiluo 2’, ‘Yuanfeng’ and ‘Xifu 2’. The Walnut Demonstration Station at Northwest A&F University in Shanyang was the location of sampling healthy and undamaged walnut fruits (33.20^o^N, 109.50^o^E). The station has an altitude of 1100 m, an annual average precipitation of 709 mm, an average temperature of 13.1 °C and an average frost-free period of 207 days. The station is located in a temperate, moist, mountainous climate zone.

### Field investigation

We observed the occurrence of walnut blight disease among the four cultivars, i.e. ‘Xiangling’, ‘Xiluo 2’, ‘Yuanfeng’ and ‘Xifu 2’ in the Shanyang Walnut Demonstration Station. In the beginning, 20 walnut trees were used for evaluation at random per cultivar and 20 fruits were selected randomly from each tree. The disease grade was determined by counting the percentage of lesions on fruits in the whole fruit area. Grade 0 had no disease; grade 1 meant lesions that accounted for less than 1/4 of the whole fruit surface; grade 2 meant lesions that accounted for 1/4 − 1/2 of the whole fruit surface; grade 3 accounted for 1/2–3/4 of the fruit surface; and grade 4 accounted for more than 3/4.

Disease index (Di) = 100 × Σ(number of diseased fruits at all grades)× (representative value at all grades)/(total number of investigated walnut fruits×4) [44].

The criteria for evaluating cultivar resistance were scaled in ranges that varied. Di ≤ 25 represented resistance, 25 < Di ≤ 40 moderate resistance, 40 < Di ≤ 55 moderate susceptibility, and Di > 55 susceptibility.

### Isolation and identification of Xanthomonas

**Fig. 14:**
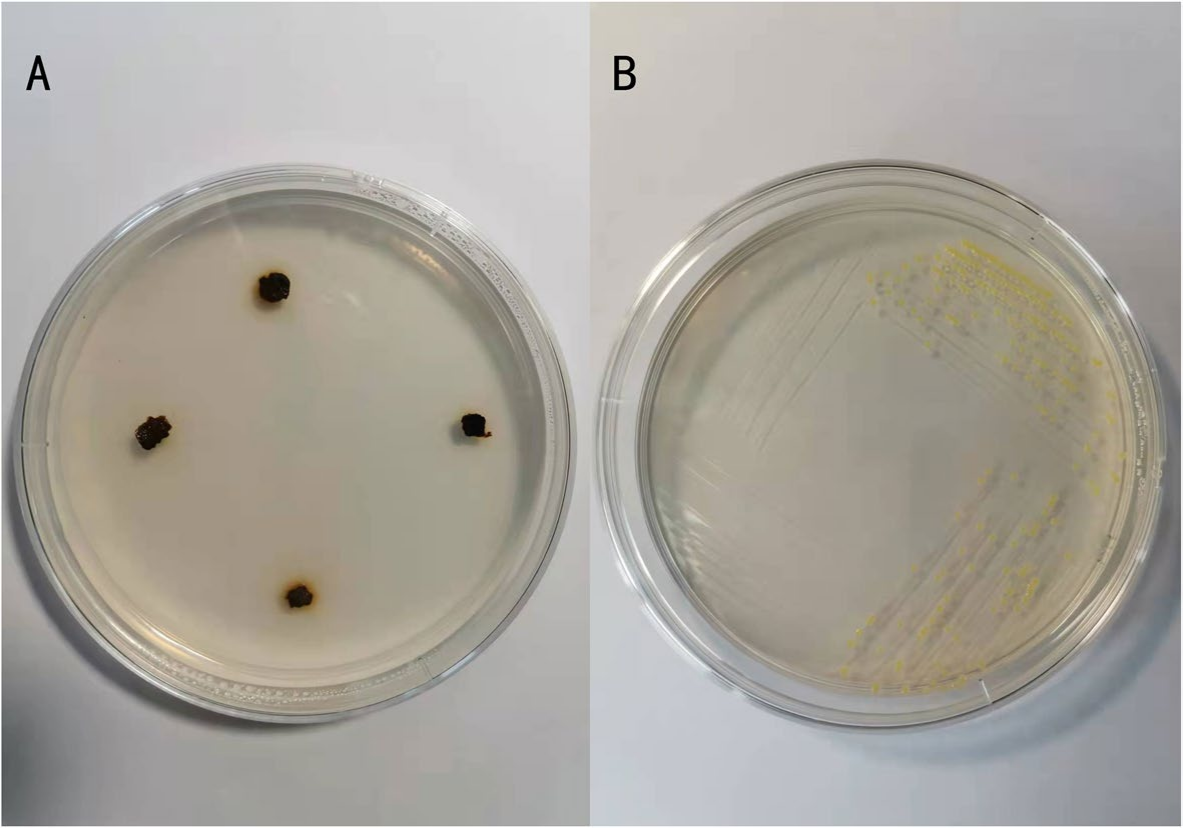
**a** Isolation of walnut blight diseased fruit; (**b**) Growth of bacteria on LB solid medium after 2 days of culture

**Fig. 15:**
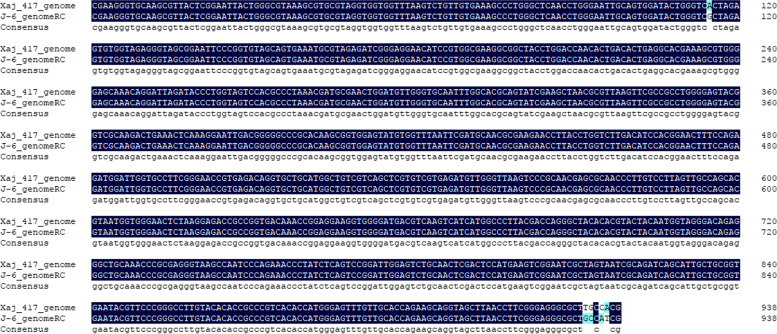
Multiple sequence alignment results

The Xaj strain was isolated and identified from disease-infected walnut fruits in Yangling, Shaanxi. First, 0.5 × 0.5 cm samples were taken from the intersection of diseased and healthy tissue. The tissue was then disinfected in 2% NaClO for 1–2 min before being rinsed in sterile water 2–3 times before drying it [45]. The fruit tissue was placed in LB solid medium at 28°C (Fig. 14a). The yellow, convex, small bacterial colonies can be observed in Fig. 14b. The DNA was extracted after a single colony was cultured in LB liquid medium. To extract bacterial DNA, the Ezup Column Bacteria Genomic DNA Purification Kit was used (Sangon Biotech, Shanghai). PCR amplification of the bacterial genome operated according to previous research by Kim et al. [46]. Based on the published 16S-rDNA universal primers in NCBI database, 27F: 5’-AGA GTT TGA TCM TGG CTCAG-3’, 1492R: 5’-GGT TAC CTT GTT ACG ACTT-3’ were synthesized by Sangon Biotech. The amplification reaction was carried out with a reaction volume of 20µL. The PCR reaction procedure was: pre-denaturation at 95℃ for 5 min, denaturation at 94℃ for 30s, annealing at 58℃ for 1 min, and extension at 72℃ for 2 min, 35 cycles. The PCR products were made on two-way sequencing in Sangon Biotech. And the sequencing results are searched for Nucleotide BLAST on the NCBI website (http://www.ncbi.nlm.nih.gov) for sequence alignment. After searching the literature and relevant data, xaj-417 was selected as the pathogenicity test strain of the walnut bacterial disease. The reference number for NCBI was CP012251.1, and the sequence homology was as high as 99.47% (Fig. 15).

### Pathogenicity determination

The Xaj-417 strain was stored at -80 °C and cultured in liquid LB medium at 28 °C. The bacterial concentration was adjusted to 2 × 10^8^ CFU mL^− 1^(OD_600_ = 0.6)in sterile water before being applied to healthy walnut fruits for their inoculation. Stab inoculation was carried out, according to Kim et al. [46], with some modifications.

We selected four cultivars, namely, ‘Xiangling’, ‘Xiluo 2’, ‘Yuanfeng’ and ‘Xifu 2’ from fruits 60 days old after pollination as the experimental material in the Laboratory of Walnut Research Center, College of Forestry, Northwest A&F University. First, we performed a 0.5 × 0.5 cm stab on the surface of fruits and there were 5 such stab areas per fruit to cause infection by bacteria in the syringe. The same fruits were treated with sterile water as the control. The filter paper was then moistened with 1 mL of sterile water in the transparent valve bag. There were 3 walnut trees per cultivar, followed by 48 fruits for each tree, a total of 192 fruits. And three biological replicates were performed for each treatment. The fruits were sampled on days 0, 4, 8, and 16 after the treatment for further analysis. During the inoculation process, we observed the phenotypic changes of walnut fruits, and the infection incidence [(number of infected fruits/total number of fruits)×100%] among different walnut varieties was counted. We also measured the lesion of diseased fruits, and the average diameter of diseased fruits was caculated to preliminarily determined the resistance of different walnut varieties. The average diameter of diseased fruits =[Σ(lesion area of each fruit per variety)]/number of diseased fruits of this variety.

### Physiological index determination

Main determinations:superoxide dismutase (SOD), catalase (CAT), peroxidase (POD), polyphenol oxide (PPO), phenylalanine ammonia lyase (PAL). And all measured enzymes were extracted from the stabbed tissue on the fruit surface of the above inoculation and control samples.

### Antioxidant enzyme extraction and activity determination

A precooled mortar containing 4 mL phosphate buffer (100 mM, pH = 7.8), 0.5% Triton-100, and 0.1 g PVPP was used for grinding the fresh walnut fruits (0.5 g). Then, the samples were ground and homogenized, while the temperature was maintained at 4 °C overnight. The solution was centrifuged for 15 min at 10,000 rpm at 4 °C [47]. The enzyme extract remained constant at 4 °C to determine the amounts of SOD, CAT and POD. Specifically, the SOD activity was determined by the NBT photoreduction method, using an amount of enzyme that could inhibit 50% of the NBT photoreduction reaction as one unit of enzyme activity. The reaction mixture consisted of phosphate buffer (50 mM pH = 7.8), 0.1 mM EDTA, 130 mM methionine, 0.75 mM NBT, 0.02 mM riboflavin [48]. CAT activity was determined according to a method by Gao [49]. Phosphate buffer (50 mM, pH = 7.0) was used in association with 200 mM H_2_O_2_. The absorbance decreased at 240 nm by 0.1 at a rate of 1 min per unit of enzyme activity. The Guaiacol method, which included phosphate buffer (200 mM, pH = 7.0), 50 mM Guaiacol solution and 0.03% H_2_O_2_ was used for determining POD activity. The reaction solution was mixed with the enzyme extract and allowed to react at 37 °C for 5 min. The absorbance at 470 nm was measured immediately after the reaction [49].

### PAL extraction and activity determination

In the PAL extraction solution, 3 mL boric acid buffer (50 mM, pH = 8.8) was used in association with 5 mM *β*-Mercaptoethanol, 1 mM EDTA and 2% PVP. With the enzyme solution (0.5 mL), 1 mL of L-phenylalanine (10 mM) and 1.5 mL of boric acid buffer (100 mM, PH = 8.8) were used for rendering the reaction operative at 37 °C for 1 h. Then, the reaction was terminated by adding 0.2 mL of HCl (6 mol·L^− 1^) and the amount of enzyme required for changing the absorbance by 0.01 (at 290 nm per hour) was defined as one unit of enzyme activity [49].

### PPO extraction and activity

The PPO solution for extraction contained 3 mL Tris buffer (50 mmol·L^− 1^, pH = 8.3), 1% (W/V) PEG 8000, 11% (V/V) glycerol, 0.015% (W/V) citric acid monohydrate and 0.010% (W/V) cysteine monohydrate, 0.01% (W/V) ascorbic acid [50]. The assay solution comprised 5 mg/mL L-Dopa in 1.4 mL, which consisted of Na_2_HPO_4_ (100 mM, pH = 7.0), 0.015% (w/v) sodium dodecyl sulfate and 280 U mL^− 1^ catalase. The absorbance was measured at 490 nm [51].

### Chemical extraction and determination

The determination method of phenolic compounds in this study was used by liquid chromatography tandem mass spectrometry. After searching the relevant literature on the content of chemical substances in walnut fruit, we finally chose to determine gallic acid, rutin, catechin, *p*-coumaric acid, chlorogenic acid, quercetin, ferulic acid, protocatechuic acid, myricetin, juglone and quercetin [52]. The standard samples in the test are all from Shanghai Yuanye Biotechnology Co., Ltd. Firstly, 10 mg of the standard was weighed and diluted to 10 mL with methanol to obtain a standard solution with a mass concentration of 1 mg/mL. And the standard curves were drawn subsequently .

Phenolic compounds were extracted from walnut fruits using an ultrasonic method and a solvent of 50% methanol (V/V) by Shi et al. [53]. Accordingly, 0.5 g stab tissue was mixed with 2 mL methanol and ground to a homogenate texture. The supernatant was centrifuged at 10,000 rpm for 30 min before being placed in a 0.5 h ultrasonic bath at 40 °C. The residue was then treated with 2 mL of methanol, and the procedure was repeated. The extracting solution was kept at 4 °C until further analysis.

Determining the TPC and TFC followed a method previously described by Skerget et al. [54] The monomer phenol was determined by passing 1 mL of the extracting solution through a 0.22 µM filter membrane and into a chromatographic flask. The HPLC analysis was performed on 1260 Infinity II HPLC System with a diode array detector (Agilent, America). The chromatographic column was the Inert Sustain SB-C18 (5.0 μm particle size, 4.6 mm×250 mm). The mobile phase was ultrapure water, with 0.1% (v/v) formic acid (eluent A) and 0.1% (v/v) formic acid acetonitrile (eluent B) [55]. All samples received an injection volume of 5 µL and a thermostat configured at 25 °C. The flow rate was 0.5 mL/min, and the gradient programs ran on a pattern of 10%~25% B (0–10 min), 25%~60%B (10–25 min), 60% B (25–35 min), and 60%~10% B (35–40 min). At 280 nm, Catechin, *p*-coumaric acid and gallic acid were detected. At 250 nm, however, protocatechuic acid, myricetin, juglone and quercetin were detected. Meanwhile, chlorogenic acid and ferulic acid were detected at 320 nm.

### Statistical analysis

For statistical analysis, the SAS (Institute, Cary, NC; 1996) statistical software package was used. The data were calculated and displayed as mean values ± SD. The data were analyzed by LSD (*p* < 0.05).

## Supplementary Information

**Additional file 1: Supplementary Figure 1.** Gallic acid content in the control (CK) and inoculated walnut fruits (Xaj) during 0-16 days. And the values are expressed as the mean ± standard deviation of three biological replicate. Letters indicated significant differences at a level of*p* < 0.05. **Supplementary Figure 2.** Catechin content in the control (CK) and inoculated walnut fruits (Xaj) during 0-16 days. And the values are expressed as the mean ± standard deviation of three biological replicate. Letters indicated significant differences at a level of* p *<0.05. **Supplementary Figure 3.** Chlorogenic acid content in the control (CK) and inoculated walnut fruits (Xaj) during 0-16 days. And the values are expressed as the mean ± standard deviation of three biological replicate. Letters indicated significant differences at a level of *p* <0.05. **Supplementary Figure 4.** *P*-coumarin acid content in the control (CK) and inoculated walnut fruits (Xaj) during 0-16 days. And the values are expressed as the mean ± standard deviation of three biological replicate. Letters indicated significant differences at a level of *p* <0.05.

## Data Availability

The datasets used and/or analyzed during the current study available from the corresponding author on reasonable request.

## Abbreviations

CAT: Catalase
Dpi: Day post infection
Na_2_HPO_4_: Dibasic sodium phosphate
EDTA: Ethylene diamine tetraacetic acid
HPLC: High performance liquid chromatography
HCl: Hydrochloride
LB: Luria-Bertani
L-Dopa: L-3,4dihydroxyphenylalanine
NBT: Nitrotetrazolium blue chloride
NADPH: Nicotinamide adenine dinucleotide phosphate
H_2_O_2_: Hydrogen peroxide
PEG: Polyethylene glycol
POD: Peroxidase
PAL: Phenylalanine ammonia lyase
PPO: Polyphenol oxidase
PVP: Polyvinyl pyrrolidone
PVPP: Crosslinked polyvinylpyrrolidone
ROS: Reactive oxygen species
RBOHs: Respiratory burst oxygenase homologs
NaClO: Sodium hypochlorite
SOD: Superoxide dismutase
TPC: Total phenol content
TFC: Total flavonoid content
Xaj: *Xanthomonas arboricola *pv. *Juglandis*
