# Supplementary material for: Protocatechuic acid, ferulic acid and relevant defense enzymes correlate closely with walnut resistance to Xanthomonas arboricola pv. juglandis
Source: BMC Plant Biol. 2022 Dec 20;22:598. doi: 10.1186/s12870-022-03997-9 (PMC9764544; doi:10.1186/s12870-022-03997-9)
Supplement: Supplementary file 1 — Additional file 1: Supplementary Figure 1. Gallic acid content in the control (CK) and inoculated walnut fruits (Xaj) during 0-16 days. And the values are expressed as the mean ± standard deviation of three biological replicate. Letters indicated significant differences at a level ofp < 0.05. Supplementary Figure 2. Catechin content in the control (CK) and inoculated walnut fruits (Xaj) during 0-16 days. And the values are expressed as the mean ± standard deviation of three biological replicate. Letters indicated significant differences at a level of p <0.05. Supplementary Figure 3. Chlorogenic acid content in the control (CK) and inoculated walnut fruits (Xaj) during 0-16 days. And the values are expressed as the mean ± standard deviation of three biological replicate. Letters indicated significant differences at a level of p <0.05. Supplementary Figure 4. P-coumarin acid content in the control (CK) and inoculated walnut fruits (Xaj) during 0-16 days. And the values are expressed as the mean ± standard deviation of three biological replicate. Letters indicated significant differences at a level of p <0.05. [file 12870_2022_3997_MOESM1_ESM.docx]

**
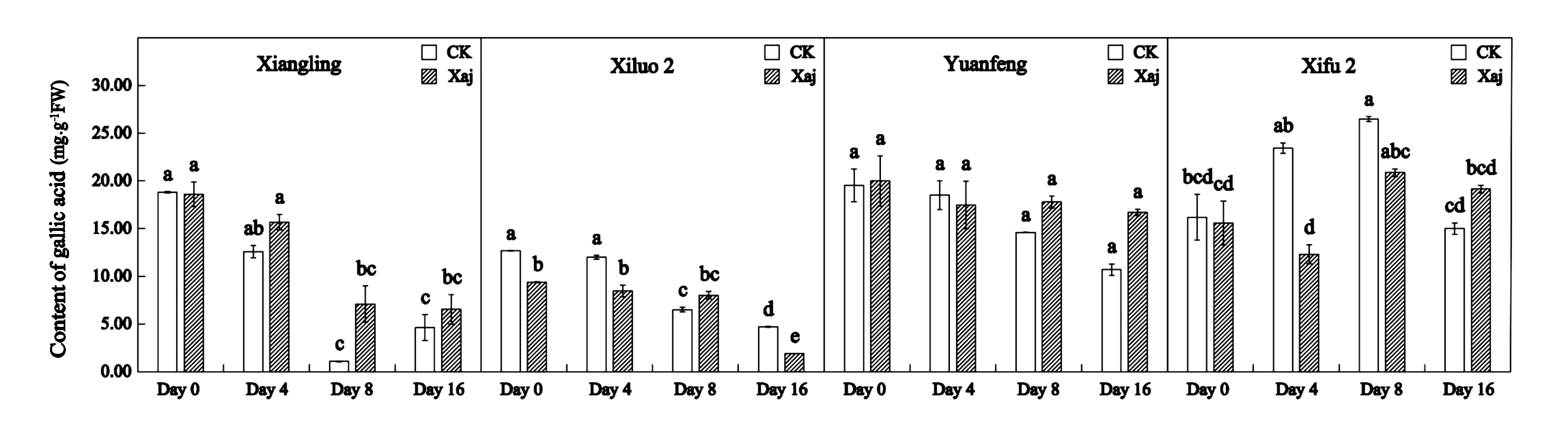
**

**Supplementary Figure 1.** Gallic acid content in the control (CK) and inoculated walnut fruits (Xaj) during 0-16 days. And the values are expressed as the mean ± standard deviation of three biological replicate. Letters indicated significant differences at a level of *p* < 0.05.


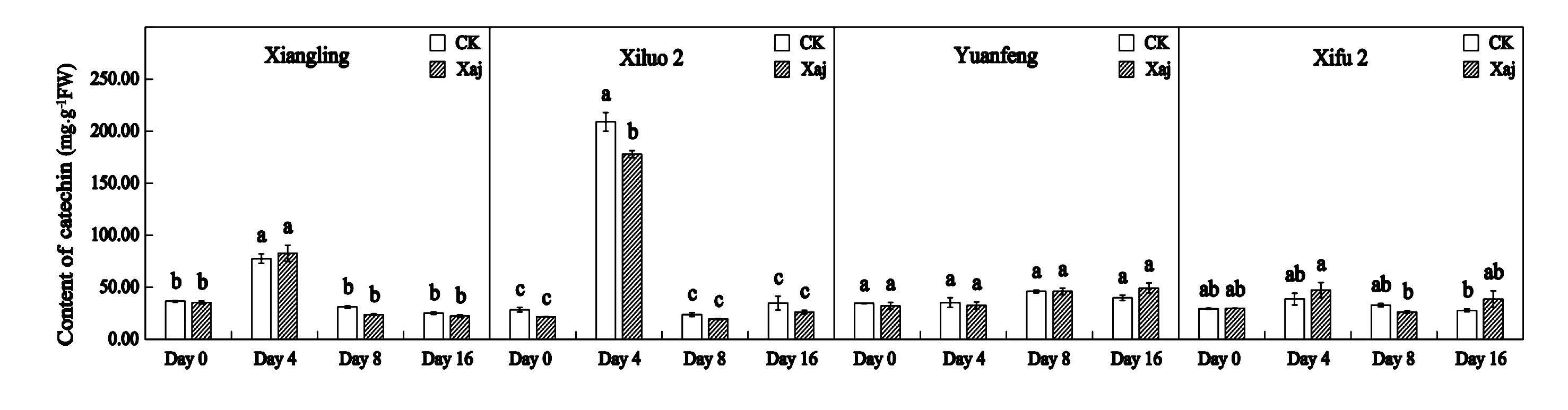


**Supplementary Figure 2.** Catechin content in the control (CK) and inoculated walnut fruits (Xaj) during 0-16 days. And the values are expressed as the mean ± standard deviation of three biological replicate. Letters indicated significant differences at a level of *p* < 0.05.


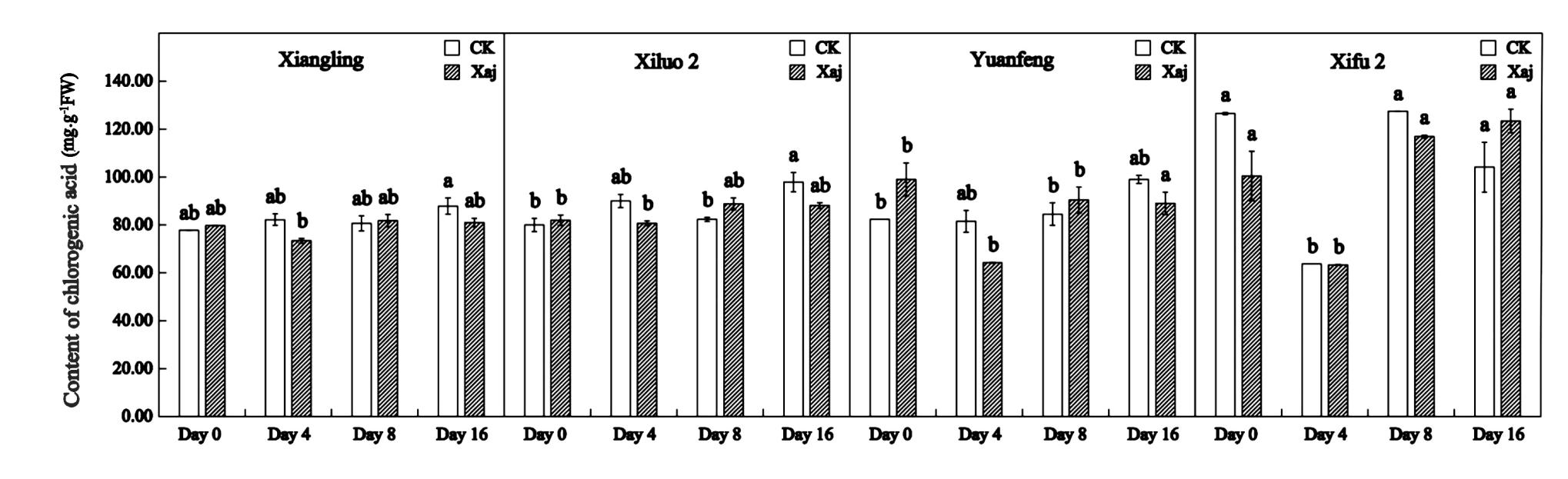


**Supplementary Figure 3.** Chlorogenic acid content in the control (CK) and inoculated walnut fruits (Xaj) during 0-16 days. And the values are expressed as the mean ± standard deviation of three biological replicate. Letters indicated significant differences at a level of *p* < 0.05.

**
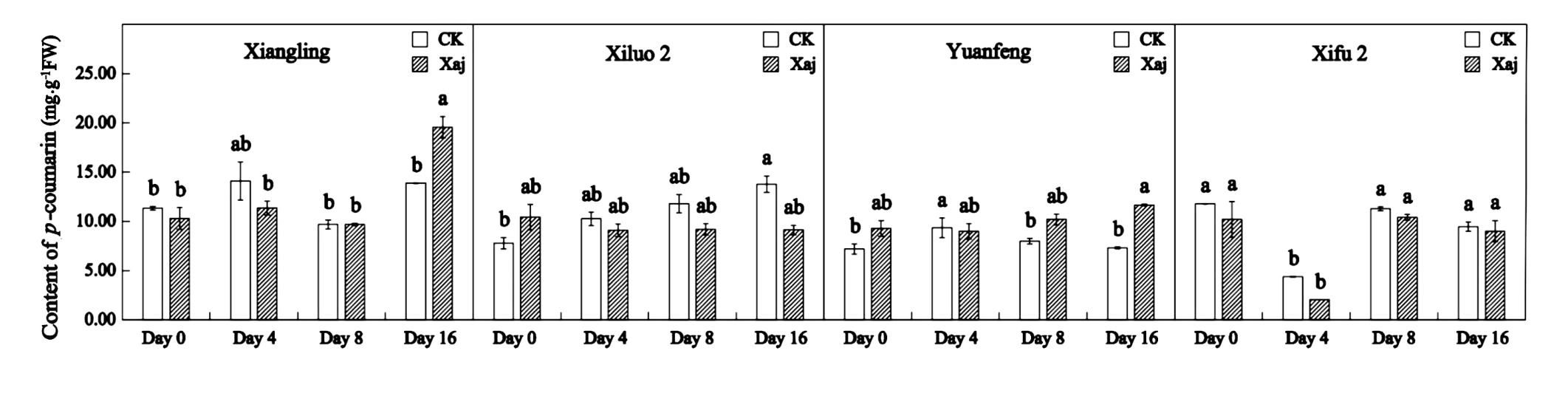
**

**Supplementary Figure 4.** *P*-coumarin acid content in the control (CK) and inoculated walnut fruits (Xaj) during 0-16 days. And the values are expressed as the mean ± standard deviation of three biological replicate. Letters indicated significant differences at a level of *p* < 0.05.
